# Anti-interleukin-6 (tocilizumab) therapy in Takayasu’s arteritis: a real life experience

**DOI:** 10.3906/sag-1906-39

**Published:** 2020-02-13

**Authors:** Levent KILIÇ, Ömer KARADAĞ, Abdulsamet ERDEN, Alper SARI, Berkan ARMAĞAN, Gözde Kübra YARDIMCI, Esra FIRAT, Umut KALYONCU, Şule APRAŞ BİLGEN, Sedat KİRAZ, İhsan ERTENLİ, Ali AKDOĞAN

**Affiliations:** 1 Division of Rheumatology, Faculty of Medicine, Hacettepe University, Ankara Turkey; 2 Department of Internal Medicine, Faculty of Medicine, Hacettepe University, Ankara Turkey

**Keywords:** Anti**-**interleukin-6, tocilizumab, Takayasu’s arteritis

## Abstract

**Background/aim:**

Tumour necrosis factor inhibitors and anti-interleukin-6 (anti-IL-6) therapies are increasingly being used in Takayasu’s arteritis (TA) patients who are unresponsive to corticosteroids ± conventional immunosuppressive agents. The aim of this study is to assess the efficacy and safety of anti-IL-6 (tocilizumab) therapy in refractory TA patients in real life.

**Materials and methods:**

Fifteen TA patients (86.7% were female) who received at least 3 cycles of tocilizumab therapy were retrospectively assessed by clinical, laboratory, and radiological evaluations before and after tocilizumab therapy.

**Results:**

The median (min–max) age of the patients at evaluation was 35 (20–58) years and the median disease duration from diagnosis was 24 (12–168) months. The median (min.–max.) duration of follow-up after tocilizumab was 15 (3–42) months. There was a significant decrease in erythrocyte sedimentation rate (ESR), C-reactive protein (CRP), and patient global visual analogue scale (VAS) scores of patients after tocilizumab therapy. The median (min.–max.) ESR was 26 (5–119) vs. 3 (2–49) mm/h, P = 0.02; CRP was 39.8 (2.4–149.0) vs. 7.9 (0–92.9) mg/L, P = 0.017; and patient global VAS was 50 (0–90) vs. 30 (0–60), P = 0.027, respectively. In 8 patients, ESR and CRP levels were in the normal range in the last control. Imaging modality results after tocilizumab were available for 9 patients; 8 patients were radiologically stable and regression was seen in 1 patient. Comparable imaging modality results before and after tocilizumab were available for 5 patients; 4 patients were radiologically stable and regression was seen in 1 patient. Radiological findings were consistent with laboratory responses. Glucocorticoid dosages decreased from a mean dosage of 16.2 (9.1) mg/day at baseline to 7.1 (3.8) mg/day (P = 0.001) at the last follow-up visit. There was no increase in the steroid dosage in any of the patients. All patients tolerated tocilizumab well.

**Conclusion:**

Based on retrospective real life data, anti-IL-6 (tocilizumab) appears to be an effective and tolerable treatment option in refractory TA patients.

## 1. Introduction

Takayasu’s arteritis (TA) is a rare chronic, inflammatory, and granulomatous type of large vessel vasculitis that predominantly affects the aorta and its main branches [1]. Although high-dose glucocorticoids are the cornerstone of the medical therapy, almost half of the patients need immunosuppressive agents due to relapses or resistance to glucocorticoids. The combinations of glucocorticoids with conventional immunosuppressive agents (methotrexate, azathioprine, mycophenolate mofetil, and leflunomide) may lead to a better response and disease control. However, clinical relapses and progression of vascular involvement remain frequent [2]. Biological therapies like TNF inhibitors (TNF-i) and anti-interleukin-6 (anti-IL-6) therapies may be effective in TA patients with uncontrolled disease [3]. IL-6, which is shown to be expressed in TA arterial lesions, influences the function of many types of cells and has a role in vascular inflammation [4–6]. The preliminary studies and case reports suggested that tocilizumab, a humanized anti-IL-6 receptor antibody, may be an option for refractory TA patients [7–15]. The aim of this retrospective observational study is to assess the efficacy and safety of tocilizumab in refractory TA patients in real life. 

## 2. Materials and methods

### 2.1. Study population

In the prospective database of the Vasculitis Centre of Hacettepe University (HUVAC), 105 TA patients meeting the 1990 modified American College of Rheumatology (ACR) criteria were registered by the end of July 2017. Totally, 28 (26.7%) patients received biological therapies and 22 (21.0%) of them were treated with tocilizumab. After reviewing the charts of these patients, 7 were excluded (2 patients had fewer than 3 cycles of tocilizumab infusion, 3 patients had no follow-up data, and 2 patients were referred to our centre after the initiation of tocilizumab). Overall, 15 TA patients who received at least 3 doses of monthly tocilizumab infusions and had available follow-up data were included. All included patients were treated with tocilizumab at 8 mg/kg intravenously every 4 weeks.

### 2.2. Demographic and clinical features

Demographic and clinical characteristics of the patients, acute phase reactants (erythrocyte sedimentation rate (ESR), C-reactive protein (CRP)), and visual analogue scale (VAS) scores for pain, fatigue, and patient global assessments were recorded at the initiation of tocilizumab therapy and at the last follow-up visit. Increased acute phase reactants were defined as ESR of >25 mm in the first hour by the Westergren method and/or CRP of >8 mg/L. A Likert scale of 0–100 mm was used for assessing VAS scores (pain, fatigue, patient global assessments). The data regarding prior and current immunosuppressive agents and concomitant glucocorticoids accompanying tocilizumab therapy were also recorded. In our cohort, some patients received alternate day prednisone (or its equivalent). Therefore, glucocorticoid dosages of patients were calculated according to the daily dosage of prednisone (or its equivalent). Available computed tomography angiography (CTA) and/or magnetic resonance angiography (MRA) results of the patients within the 3 months prior to tocilizumab initiation and during follow-up were obtained from the hospital data system. According to the angiographic findings, TA patients were classified into 5 categories [16].

### 2.3. Laboratory and radiological evaluations

Patients’ clinical, laboratory, and radiological data and treatments were analysed at baseline (at the initiation of tocilizumab) and the last follow-up visit. The radiological evidence of new areas of vessel involvement or worsened vascular lesions was considered as an ‘active’ disease. The radiological disease activity was classified as stable disease (absence of any change in lesions), progression (development of a new lesion: vessel wall thickening/irregularity, stenosis, occlusion, aneurysm, and dilatation), and regression (decline in existing lesions). Patients were considered to have ‘clinically active’ disease if they had persistence or exacerbation of the clinical symptoms and elevated inflammatory laboratory markers despite unchanged angiographic findings. The adverse events attributable to tocilizumab, such as infusion reactions, infections requiring hospitalization, elevated liver enzymes, hypertriglyceridemia, and leukopenia, were checked for safety assessment.

### 2.4. Ethical considerations

The Turkish Ministry of Health granted the compassionate use of anti-IL-6 for TA patients and every patient planned to undergo this treatment signed an informed consent form. Therefore, this study was performed after the approval of both the local ethical committee (approval number: GO 18/266-01) and the Turkish Medicines and Medical Devices Agency of the Turkish Ministry of Health (approval number: 75642246-000-E.107089)

### 2.5. Statistical analysis

Statistical analysis was performed using SPSS 21.0 (IBM Corp, Armonk, NY, USA). The values are expressed as mean ± SD and median (min–max), unless indicated otherwise. The Wilcoxon signed rank test was used to compare the parameters whenever appropriate. P < 0.05 was considered as statistically significant.****

## 3. Results

Fifteen patients (86.7% females) who received at least 3 doses of monthly tocilizumab infusions and had available follow-up data were included in the analysis. The median (min–max) age of the patients was 35 (20–58) years and the median disease duration from diagnosis was 24 (12–168) months. The median (min–max) follow-up for tocilizumab therapy was 15 (3–42) months. The clinical and demographic characteristics of the patients are shown in Table 1. Before tocilizumab therapy, all patients received corticosteroids and at least 1 conventional immunosuppressive agent [12 (80%) patients received methotrexate, 7 (46.7%) patients received intravenous cyclophosphamide, and 4 patients received azathioprine, leflunomide, or mycophenolate mofetil]. Also, 5 (33.3%) patients received TNF-i before tocilizumab (Table 2). The indications for tocilizumab therapy were as follows: radiologically active disease in 6 patients (acute phase reactants were normal in one of them) and clinically active disease in 9 patients (5 of them were radiologically stable and the other 4 patients had no radiological imaging). 

**Table 1:** The clinical and demographic characteristics of the TA patients.

Patient no.	Age/ sex	Age at diagnosis	Disease duration (months)	Type of vascular involvement*	Comorbidity
1	57/F	53	48	2a	Hypothyroidism
2	40/M	31	72	2a, P(+)	IBD, EpA
3	47/F	37	96	2b	AS, OP
4	25/F	20	36	5, P(+)	HT
5	55/F	53	12	5	HT, RA
6	40/F	36	12	5	HT, AS
7	30/F	27	12	2b	HT
8	28/F	23	48	2b, C(+)	AVR
9	52/F	35	168	5	-
10	43/F	40	24	5, P(+)	HT
11	31/F	23	84	5, P(+)	HT
12	31/F	29	24	5	HT
13	59/F	58	12	5	OP
14	49/F	46	12	5	HT
15	24/M	20	24	2a, P(+)	-

F: Female, M: Male, P: Pulmonary involvement, C: Coronary involvement, IBD: Inflammatory bowel diseases, EpA: Enteropathic arthritis, AS: Ankylosing spondylitis, HT: Hypertension, RA: Rheumatoid arthritis, AVR: Aortic valve replacement, OP: Osteoporosis. *According to the angiographic findings, TA patients were classified into 5 categories [16].

**Table 2:** The parameters of disease activity before and after tocilizumab therapy.

	Before tocilizumab therapy						After tocilizumab therapy						
Patient no	Previous treatments	Steroiddosage	ESR mm/s	CRPmg/L	VAS (0–100)(Pain/fatigue/pt. global)	Imaging	Follow-upduration(months)	ESRmm/h	CRPmg/L	Steroid dosage	VAS (0–100)(Pain/fatigue/pt. global)	Imaging	Outcome/adverseevents
1	MTX	Pred. 15mg/ad	34	42.80	0/20/20	MRA:Stable	3	3.00	2.00	Pred. 15 mg/ad			
2	CYC, MTX, SSZ, IFX, ADA	Pred. 10mg/day	75	81.70	-	MRA:Stable	34	49.00	92.90	Pred. 5 mg/day		MRA:Stable	
3	CYC, SSZ, MTX, PS, ETN	MPZ 32mg/ad	24	19.6	50/70/30	MRA: Progression	18	12.00	18.10	MPZ 12 mg/ad	30/60/30		
4	CYC, MTX, LEF, IFX, ADA	Pred. 30mg/ad	11	42.7	80/70/60		20	20.00	16.50	Pred. 20 mg/ad	20/50/20	CTA:Stable	
5	MMF	MPZ 16mg/day	68	76.1	-	MRA:Stable	5	30.00	80.00	MPZ 4 mg/day			*Switched to adalimumab
6	MTX, PS, IFX	Pred. 40mg/ad	26	28.1	-		28	2.00	1.70	Pred. 30 mg/ad			*Switched to adalimumab
7	MTX, PS, IFX	Pred. 40mg/ad	119	149.0	70/50/70	BTA: Progression	21	2.00	1.70	Pred. 30 mg/ad	30/30(30	MRA:Stable	Fatigue
8	MTX, PS	PS 500mg/month	103	85.1	0/0/0		15	12.00	37.50	PS 500 mg/month	0/0/0	CTA:Stable	
9	CYC. MTX	Pred. 40mg/ad	35	63.7	10/30/40		42	23.00	16.20	Pred. 5 mg/ad	10/20/30	MRA:Stable	Dizziness
10	CYC, MTX	-	12	16.7	90/90/90	BTA: Progression	10	2.00	1.20	-		CTA:Regression	
11	CYC, MTX	Pred. 20mg/ad	6	2.7	40/60/30	MRA: Progression	6	2.00	1.90	Pred. 15 mg/ad		MRA:Stable	
12	AZT, MTX	Pred. 30mg/ad	7.00	2.4	40/70/60	MRA:Stable	3	2.00	5.10	Pred. 15 mg/ad	40/70/60		
13	CYC,	Pred. 5mg/day	42.00	39.8	70/60/70	BTA:Stable	3	3.00	1.00	-	50/50/50		
14	MMF	MPZ 6mg/day	5.00	8.6	50/80(80	MRA: Progression	7		10.50	MPZ 4 mg/day	60/60/60	MRA:Stable	Leg swelling
15	CYC, MTX	Pred. 40mg/day	16.00	31.70	0/0/10	BTA: Progression	27	2.00	7.90	Pred. 12.5/day	10/0/5	MRA:Stable	

CYC: Cyclophosphamide, MTX: Methotrexate, SSZ: Sulfasalazine, LEF: Leflunomide, MMF: Mycophenolate Mofetil, AZT: Azathioprine, PS: Pulse steroid, IFX: Infliximab, ETN: Etanercept, ADA: Adalimumab, Pred.: Prednisolone, MPZ: Methylprednisolone, ad: on alternate days, MRA: Magnetic resonance angiography, CTA: Computed tomography angiography. *Tocilizumab was switched to adalimumab in 2 patients, due to ineffectiveness in P5 and the activation of accompanying ankylosing spondylitis in P6.

Tocilizumab was combined with methotrexate in 8 patients and in 1 patient with leflunomide. There was a significant decrease in median (min–max) ESR [26 (5–119) mm/h vs. 3 (2–49) mm/h, P = 0.02], CRP [39.8 (2.4–149.0) mg/L vs. 7.9 (0–92.9) mg/L, P = 0.017], and patient global VAS assessment [50 (0–90) vs. 30 (0–60), P = 0.027] after tocilizumab. In 8 patients, ESR and CRP levels were in the normal range at the last follow-up visit. During follow-up, tocilizumab was ceased in 2 patients due to ineffectiveness in 1 patient (P5) and activation of accompanying ankylosing spondylitis in the other (P6). Control vascular imaging studies after tocilizumab therapy were available for 9 patients, while 8 patients were radiologically stable and regression was seen in 1 patient compared to the last imaging before tocilizumab. However, 4 radiologically stable patients had CRP levels above the upper limit of normal (another patient (P2) who was radiologically stable but had increased acute reactants had concomitant active AS). The mean daily dose of prednisone (or its equivalent) at the first tocilizumab administration was 16.2 (9.1) mg/day while the mean dosage at last follow-up was 7.1 (3.8) mg/day, showing that there was a significant decrease in glucocorticoid dosage after tocilizumab (P = 0.001). During the follow-up, no serious adverse events such as severe infections, gastrointestinal perforations, or infusion related reactions occurred. We did not observe leukopenia or neutropenia in any of the patients. There were minor nonspecific side effects in 3 patients (malaise in 1 patient, leg swelling in 1 patient, and dizziness in another patient) without causing drug cessation.

## 4. Discussion

This study is one of the largest series evaluating the effect of tocilizumab therapy in refractory TA patients in real life. Our findings supported the effectiveness of tocilizumab in the treatment of refractory TA without major adverse events. In most patients, acute phase reactant levels were decreased and follow-up imaging findings were stable. 

 Previously, several case reports and observational studies done with small groups of patients reported the efficacy and safety of tocilizumab in refractory TA, including the patients unresponsive to TNF-i [7–15]. Recently, Decker et al. published 4 cases and an updated literature review on the tocilizumab efficacy and safety in patients with TA [17]. In this literature review, among 105 patients, most cases were refractory and 90 patients (85.7%) had an initial clinical meaningful response within 3 months. Among these patients, 17 (77%) of 22 patients who were previously treated with TNF-i had clinical improvement with tocilizumab. A corticosteroid dose reduction was achieved in 75 of the 83 patients (90.4%). The CRP and ESR levels at the end of follow-up were reduced compared to the initiation of tocilizumab treatment. Radiological improvement was noted in 43 of the 66 patients (65.2%). In that study, only 7 patients were considered to have relapse in a median treatment duration of 12 months [17]. Recently, the efficacy and safety of tocilizumab (162 mg/week subcutaneously) was investigated in a randomized, double-blind, placebo-controlled, and phase-3 trial (the TAKT study) from Japan [18]. For inclusion, patients had to be receiving a stable glucocorticoid dosage at least twice the dose at relapse and be in remission for 1 week. Thirty-six patients with TA were randomized 1:1 to receive weekly tocilizumab (162 mg/week subcutaneously) or its placebo. In both groups, prednisone was tapered by 10%/week from week 4. The primary endpoint was time to relapse based on the signs and symptoms of TA without imaging evaluation. The primary endpoint was not met in this study. However, a treatment difference suggesting favour for tocilizumab was observed in the per-protocol set sensitivity analysis and secondary endpoints. However, the results of this study should be interpreted keeping in mind that it includes only refractory TA patients and a low number of patients [18]. The responses to tocilizumab therapy in our study are consistent with the previous reports in the literature. There was a significant decrease in glucocorticoid dosage and ESR and CRP levels, and most of the patients’ radiological findings were stable after tocilizumab therapy. Decker et al. reported that 18 (18%) out of 101 patients had adverse effects with tocilizumab therapy (10 infections, 5 cytopenia, 6 hepatitis, 1 pancreatitis, 1 cutaneous rash, and 1 breast cancer) and 7 (7%) patients’ therapies were stopped due to the adverse effects [17]. In our study, tocilizumab was well tolerated by all of the patients. Minor side effects which were nonspecific and did not cause drug cessation were observed in 3 patients. Tocilizumab therapy was switched due to ineffectiveness in 1 patient and in another due to the activation of accompanying ankylosing spondylitis. Although we reported a good clinical and radiological response in refractory TA patients with tocilizumab, our results should be evaluated with caution. Firstly, this was a retrospective study with a limited number of patients. Secondly, we used the clinical and laboratory parameters to define the disease activity. Evaluation of the disease activity of TA is still challenging. Previous studies showed a progression of vascular lesions and the presence of histologically active disease in half of the patients despite clinical and laboratory remissions [19,20]. Thirdly, TA is generally a slowly progressive disease and the progression rates of radiological findings are variable [1,19]. Therefore, although we did not document radiological progression of any patients, treatment duration in our study may not be sufficient to make a final conclusion. 

Increasing importance of IL-6 in the pathogenesis of TA promises the use of tocilizumab in refractory TA. Our results support the clinical effectiveness of tocilizumab in this patient group. However, long standing studies with sufficient numbers of patients are needed to document the effectiveness and safety of tocilizumab. 

## Acknowledgement/Disclaimers/Conflict of interest

The author(s) declared no potential conflicts of interest with respect to the research, authorship, and/or publication of this article. The author(s) received no financial support for the research, authorship, and/or publication of this article.

## Informed consent

All patients gave written informed consent. The study protocol was approved by the Local Research Ethics Committee (GO-15/747).
